# Three-dimensional evaluation of bracket placement accuracy and excess bonding adhesive depending on indirect bonding technique and bracket geometry: an in-vitro study

**DOI:** 10.1186/s13005-020-00231-5

**Published:** 2020-08-03

**Authors:** Stephan Christian Möhlhenrich, Constantin Alexandridis, Florian Peters, Kristian Kniha, Ali Modabber, Golamreza Danesh, Ulrike Fritz

**Affiliations:** 1 Department of Orthodontics, University of Witten/Herdecke, Alfred-Herrhausen Str. 45, 58455 Witten, Germany; 2 Department of Orthodontics and Dentofacial Orthopedics, University Hospital of the RWTH Aachen, Pauwelsstraße 30, 52074 Aachen, Germany; 3 Department of Oral and Maxillofacial Surgery, University Hospital of the RWTH Aachen, Pauwelsstraße 30, 52074 Aachen, Germany

**Keywords:** Indirect bonding, transfer accuracy, Siloxane tray, Bracket, excess adhesive

## Abstract

**Background:**

This study aimed at comparing bracket placement and excess bonding adhesive depending on different indirect bonding (IDB) techniques and bracket geometries.

**Methods:**

Four hundred eighty brackets without hook (WOH) and 360 with hook (WH) were placed on 60 plaster models. Three IDB techniques were tested: polyvinyl-siloxane vacuum-form (PVS-VF), polyvinyl-siloxane putty (PVS-putty), and translucence double-polyvinyl-siloxane (double-PVS). PVS-VF and PVS-putty were combined with chemically, and double-PVS was combined with light cured bonding adhesive. Virtual images of models before and after bracket transfer were generated, and computerized images were compared. Linear, angular deviations, and excess bonding adhesive were measured.

**Results:**

Linear differences between the three groups were obtained for PVS-VF (WH: 1.08, SD 0.50 mm; WOH: 0.86, SD 0.25 mm), PVS-putty (WH: 0.73, SD 0.51 mm; WOH: 0.58, SD 0.28 mm), and double-PVS (WH: 0.65, SD 0.45 mm; WOH: 0.59, SD 0.33 mm) (*P* < 0.001). Hooks affected bracket placement accuracy in PVS-VF (*P* < 0.001) and PVS-putty (*P* = 0.029). Angular differences were observed for brackets WOH between the PVS-VF (0.64, SD 0.48°) and double-PVS group (0.92, SD 0.76°) (*P* < 0.001) and within double-PVS group (WH: 0.66, SD 0.51° vs. WOH: 0.92, SD 0.76°, *P* < 0.001). Highest amount of excess adhesive was obtained for PVS-putty group (WH: 6.54, SD 5.31 mm ^2^).

**Conclusions:**

The double-PVS group revealed promising results with respect to transfer accuracy, whereas the PVS-VF group provided least excess bonding adhesive. Basically, hooks lead to lower precision and higher excess bonding adhesive. PVS trays for IDB generate high bracket placement accuracy. PVS-putty is the easiest to handle with and also the cheapest, but leads to large excess bonding adhesive, especially in combination with hooked brackets or tubes.

## Background

Orthodontically treated fixed straight-wire appliances allow three-dimensional tooth movements. To reduce the need for arch wire bends or bracket repositioning, brackets should be ideally placed. Four parameters must be considered for achieving the ideal bracket position [1]: 1) bracket base adaptation to the tooth surface contour, 2) evaluation of the rotational position of each bracket from the occlusal direction, 3) determination of the vertical position of each bracket, and 4) determination of the desired slot angulations of each bracket by evaluating root position [2].

Silverman et al. was the first to introduce the indirect bonding (IDB) technique [3]. For this purpose, brackets were ideally placed on dental casts to be transferred to the patient’s tooth using a fabricated IDB tray. The benefits of this technique are the unimpaired bracket positioning visibility, improved patient comfort, and reduced chair time [3–7]. Several studies have reported that in general, IDB trays lead to higher bracket placement accuracy than the usually used direct bonding techniques [4, 6, 8–11]. This is attributed to the fact that in the first stage, the placement of the attachments is completed away from clinical influences and variables that complicate the direct method, such as moisture control, patient management, or hurried schedules [12]; however, the placement of the brackets on the dental cast also includes potential constraints and variables, which can influence the reliability of the transfer to the patient’s dentition. Possible influencing factors, which affect final bracket placement error during transfer, can be errors in tray fabrication, contaminants or soft-tissue interferences, bonding thickness, and adhesive material between the brackets and teeth during clinical bonding as well as errors in clinical technique. Thus, differences in bracket transfer accuracy as a function of trays and tooth type were already reported [12, 13]. Current studies on IDB tray-dependent bracket accuracy focus on vacuum-formed thermoplastic sheets, silicone materials, or a combination of both. Dorfer et al. compared bracket transfer accuracy between three different IDB techniques and found that bracket transfer accuracy was significantly better for trays made of addition silicone than single vacuum-formed trays [14]. Castilla et al. also obtained similar results, wherein five transfer techniques were compared with each other and overall small differences in bracket position were observed; however, the silicone-based trays had a highly consistent high transfer bracket accuracy, whereas methods that exclusively used vacuum-formed trays were less consistent [13]. In this context, the possible influence of the bracket geometry itself, such as hook presence, has not been investigated yet.

Another important aspect of bracket attachment is excess bonding adhesive [15]. A sufficient marginal seal and less bonding material around the bracket are necessary to avoid caries or white-spot lesions [16]. Particularly, the transition between orthodontic adhesive and enamel surrounding the tooth structure of the bracket base is a prerequisite for demineralization during orthodontic treatment [17, 18]. If this is not adequately removed, the rough surface of the remaining bonding adhesive provides a site for the rapid attachment and growth of oral microorganisms, finally resulting in plaque accumulation [19, 20]. Furthermore, acid-rich cariogenic dental-plaque biofilm conditions could result in deterioration of the bonding and adhesive material in the cured adhesive layer because of well-delineated pockets and air channels [21]. Therefore, residual adhesive bonding material must be avoided or immediately removed after attachment positioning and before material curing. However, this is not possible when using IDB techniques because the transfer tray hampers access to the area around the bracket.

The present study aimed at investigating the influence of three different three-dimensional IDB techniques, as well as the influence of bracket geometry on bracket placement accuracy and excess bonding material. The first hypothesis was that neither the different transfer techniques nor the presence of hooks on the brackets would lead to differences between the working and transfer models of bracket position. Furthermore, the second hypothesis was that there are also no differences in the excess bonding adhesive depending on IDB technique or bracket geometry.

## Methods

**Fig. 1:**
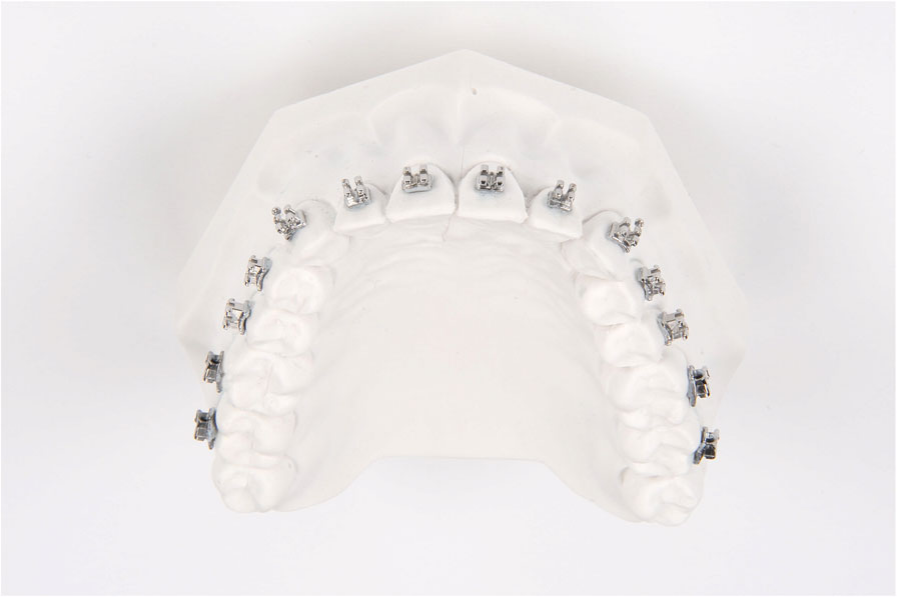
Working model of a maxillary typodont with adhesive pre-coated brackets and tubes in ideal teeth positions

**Fig. 2:**
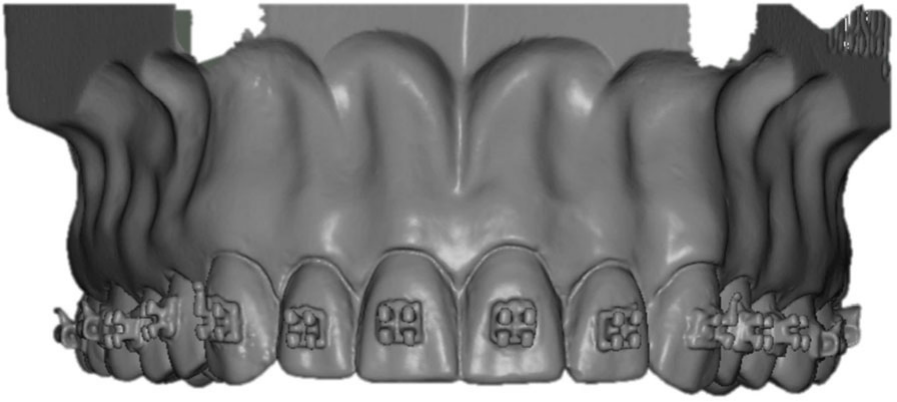
3D working model of a maxillary typodont with placed brackets and tubes

Sixty plaster models (plaster type IV, Rapidur®, Dentaurum, Ispringen, Germany) of a maxillary as well as mandibular typodont (Model G1 and G2, Frasaco GmbH, Tettnang, Germany) were prepared. Thirty maxillary and mandibular models (10 per group) served as working models, and 30 served as transfer models. The working models were coated with a separating agent (Bioplast, Scheu Dental GmbH, Iserlohn, Germany). After drying, adhesive pre-coated (Greengloo, Ormco, Orange CA, USA) brackets and tubes (discovery® smart, Mclaughlin-Bennett-Trevisi 22, Ortho-Cast M-Series 22, Dentaurum, Ispringen, Germany) were placed and adhesive excesses around the bracket were removed. Then the brackets were light-cured in ideal positions (bracket base point positioned over the facial axis and aligned parallel to the clinical crown) on incisors, canines, premolars, as well as first and second molars (Fig. 1). The brackets were subsequently coated with scanspray (Cerec Optispray, Sirona Dental System GmbH, Bensheim, Germany), and the models were transferred to virtual reality using digital scans generated by a 3D model scanner (orthoX® scan, Dentaurum, Ispringen, Germany) (Fig. 2).

### Indirect bonding trays

**Fig. 3:**
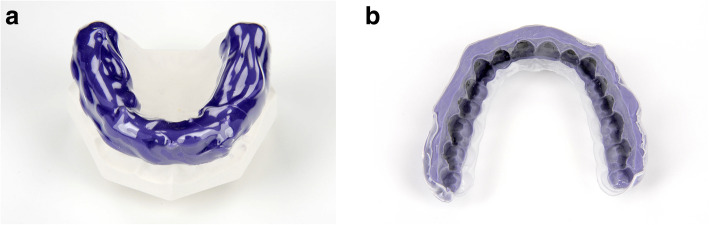
**a** and **b**: PVS-VF transfer tray: polyvinyl siloxane and clear vacuum-formed 0.5 mm polyethylene terephthalate glycol (PETG) sheet

**Fig. 4:**
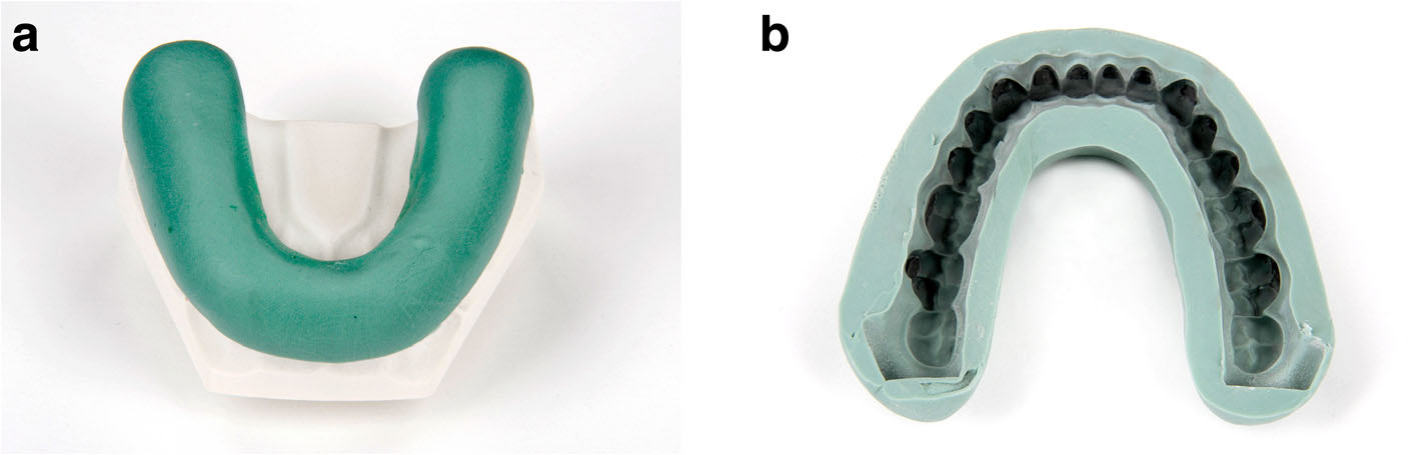
**a** and **b**: PVS-putty transfer tray: high-viscosity polyvinyl siloxane putty

**Fig. 5:**
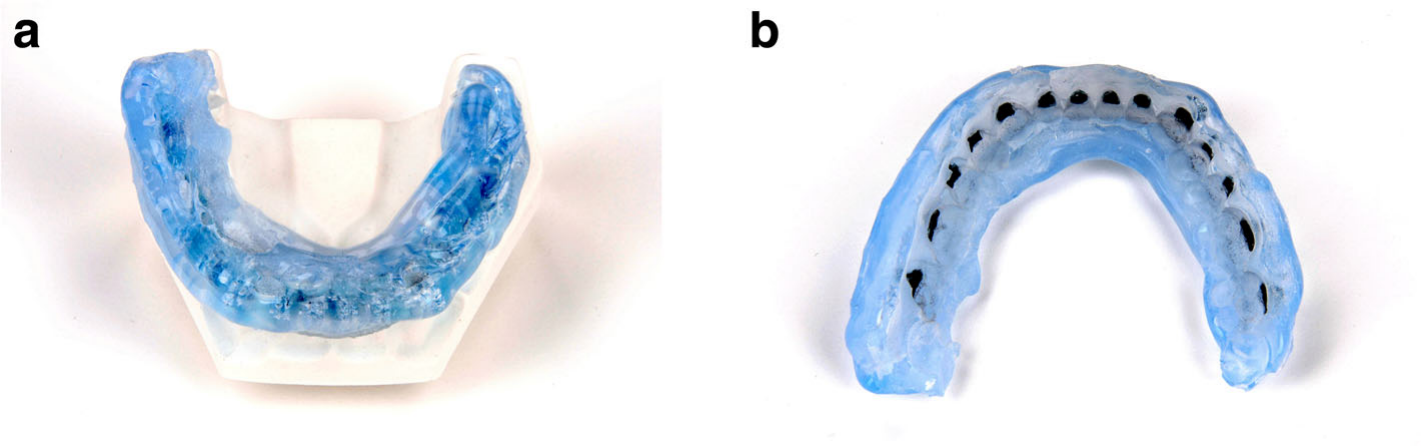
**a** and **b**: Double-PVS transfer tray: clear soft silicone and clear polyvinyl siloxane

Three different IDB trays were fabricated for their corresponding working models (Table 1): group 1 (PVS-VF)- polyvinyl siloxane and clear vacuum-formed 0.5 mm polyethylene terephthalate glycol (PETG) sheet (S4 suhy light, bisico, Bielefeld Germany; Duran, Scheu Dental GmbH, Iserlohn, Germany) (Fig. 3 a and b), group 2 (PVS-putty)- very high-viscosity polyvinyl siloxane putty (S1 suhy, bisico, Bielefeld Germany) (Fig. 4 a and b), group 3 (double-PVS)- clear soft silicone and clear polyvinyl siloxane (Emiluma & Lumaloc, Opal Orthodontics, Ultradent, South Jordan, UT, USA) (Fig. 5 a and b). Similar to the previous publications, all transfer trays covered the buccal, occlusal, and lingual surfaces [4, 13, 22].

**Table 1:** Descriptions for used indirect bonding protocols

Indirect Bonding Technique	Tray Material		Bonding adhesive
	Single/Inner	Outer	
PVS-VF	Light-body polyvinyl siloxane (S4 suhy light, bisico, Bielefeld Germany), faciolingual thickness: 3 mm	Clear vacuum-formed 0.5-mm polyethylene terephthalate glycol (PETG) sheet (Duran, Scheu Dental GmbH, Iserlohn, Germany)	•Pre-Coting: Greengloo, Ormco, Orange CA, USA •Two-part chemically cured bonding adhesive (Maximum Cure Sealant Part A and B, Reliance Ortho Prod. Inc., Itasca, IL, USA)
PVS putty	Very high viscosity polyvinyl siloxane putty (S1 suhy, bisico, Bielefeld Germany), faciolingual thickness: 5–6 mm		•Pre-Coting: Greengloo, Ormco, Orange CA, USA •Two-part chemically cured bonding adhesive (Maximum Cure Sealant Part A and B, Reliance Ortho Prod. Inc., Itasca, IL, USA)
Double-PVS	Clear soft silicone (Emiluma, Opal Orthodontics, Ultradent, South Jordan, UT, USA), faciolingual thickness: 1–2 mm	Clear polyvinyl siloxane (Lumaloc, Opal Orthodontics, Ultradent, South Jordan, UT, USA), faciolingual thickness: 5–6 mm	•Pre-Coting: Greengloo, Ormco, Orange CA, USA •Light cured bonding adhesive Gluma Solid Bond S, Heraeus Kulzer GmbH, Hanau, Germany), low viscosity composite (Venus Flow, Heraeus Kulzer GmbH, Hanau, Germany)

Before removal from the working models, the trays were soaked in water to remove any separating agent and were finally cleaned and sandblasted according to Sondhi et al. [22].

### Bonding adhesive

In group 1 (PVS-VF) and 2 (PVS-putty), a two-part chemically cured bonding adhesive was used for IDB (Maximum Cure Sealant Part A and B, Reliance Ortho Prod. Inc., Itasca, IL, USA). Sealant part A was applied to the individual composite bracket base and part B to the transfer models. Each tray was equally seated with determined pressure by one investigator with both hands. After 3 min, the bonding adhesive was cured and the trays were removed from the lingual to the buccal side of the teeth. In group 3 (double-PVS), a light-cured bonding adhesive was used for IDB (Gluma Solid Bond S, Heraeus Kulzer GmbH, Hanau, Germany). After adhesive bonding, a low viscosity composite was applied on the individual composite bracket base (Venus Flow, Heraeus Kulzer GmbH, Hanau, Germany) and trays were seated with determined pressure on the transfer models. Each tooth was subsequently exposed to LED light for 20 s, and the trays were finally removed from the lingual to the buccal side of the teeth. After tray removal, the brackets were again sprayed with scanspray and transferred to virtual reality using the 3D model scanner.

### Virtual measurements

**Fig. 6:**
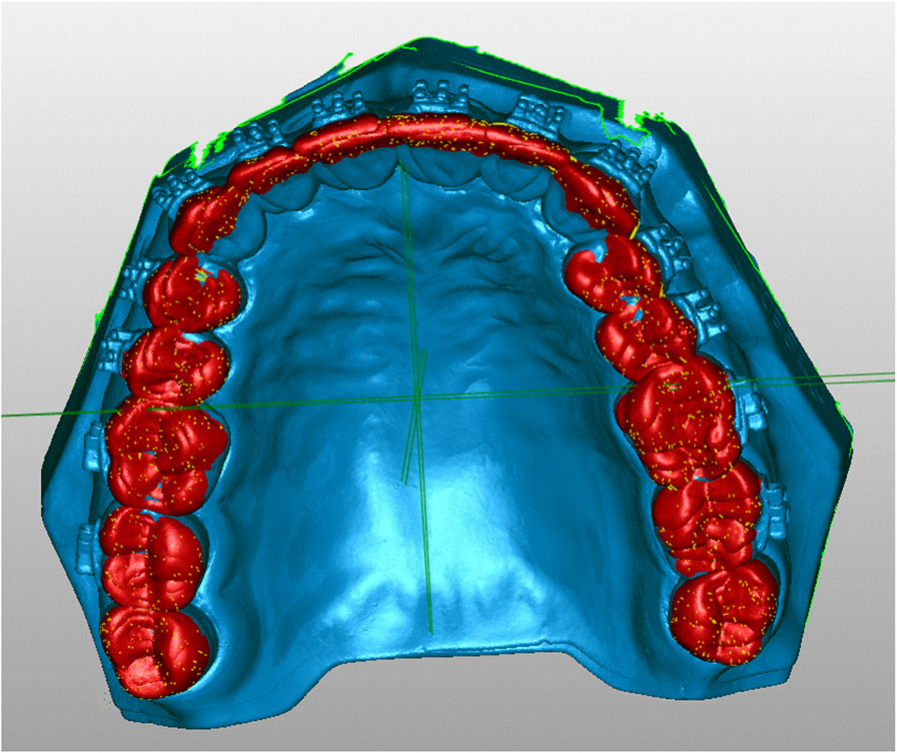
Automatic surface registration of the virtual models on the basis of an iterative closest point algorithm with occlusal surface and incisal edge of the front teeth as reference areas

**Fig. 7:**
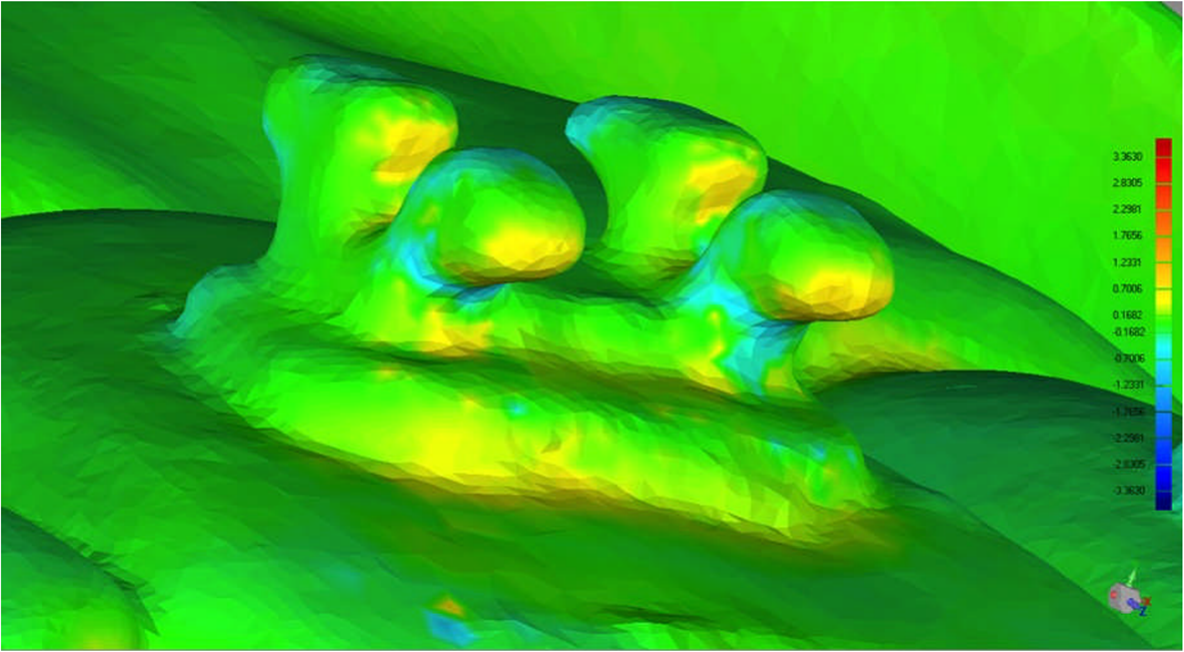
Software based comparison of the virtual models before and after bracket transfer using an iterative closest point algorithm, green indicates ideal proximity of objects, red indicates increased differences and blue indicates decreased differences

**Fig. 8:**
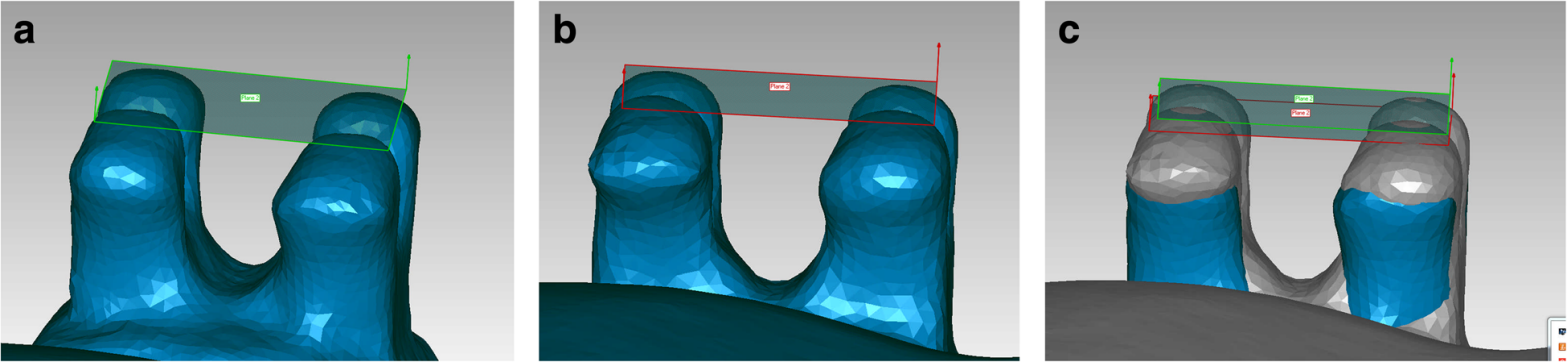
**a-c** Determination of the maximum angle deviation, **a** Reference plane at the level of the four wings (working model), **b** Reference plane at the level of the four wings (test model), **c** Superimposition of reference and test model with corresponding planes for measuring the angle in between

**Fig. 9:**
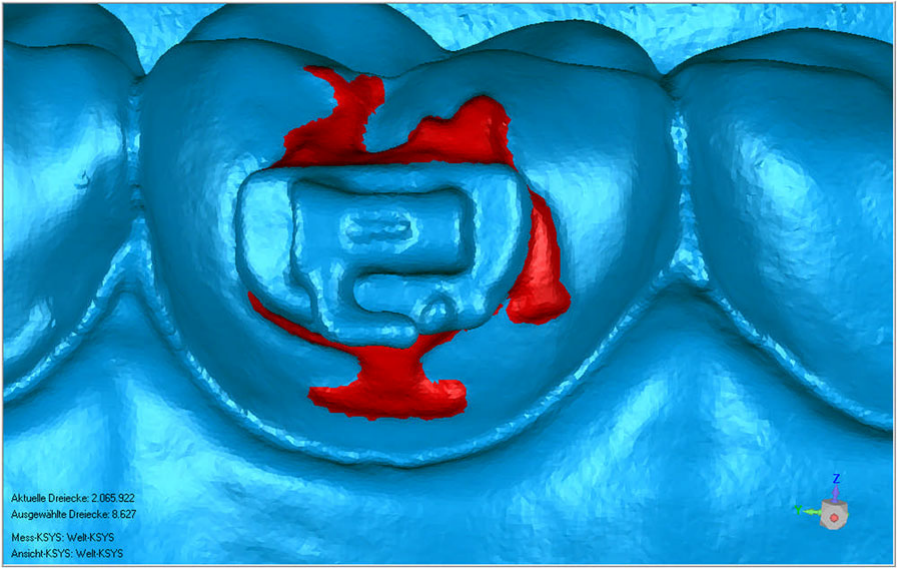
Excess bonding adhesive after bracket transfer surrounding a tube

Stereolithographic files were imported into the Geomagic Qualify software (Geomagic, Morrisville, NC, USA). Using automatic surface registration of the virtual models on the basis of an iterative closest point algorithm, working and transfer models were compared (Fig. 6). Therefore, the occlusal surface and incisal edge of the teeth were chosen as reference areas. The working model served as the reference model, and the transfer model served as the test model. The brackets placed on the buccal side of the teeth were set as the region of interest. Maximum linear deviations, maximum angular deviations, and excess adhesive surrounding the brackets were measured. The measured maximum deviation indicates the maximum Euclidean distance between two corresponding points of a certain bracket, which were set as “test” to the same point on the object set as “reference”, when testing the whole bracket surface. The average measured deviation resulted of these maximum measurements for each bracket with and without hook. This completely automated process leads to no method error by the investigator and generates a full-color deviation map and histogram comparing the two surfaces. The color map overlay shows the proximity of objects in green, whereas red represents increased differences in the distance from the virtual simulation (Fig. 7). Maximum angular deviation is defined as the greatest possible angle between reference and test plane applied to the four wings of each bracket (Fig. 8) and excess adhesive results from the area of color map surrounding the bracket (Fig. 9).

### Statistical analysis

For each parameter, a mixed-model measure ANOVA was performed to examine the influence and possible interaction between the IDB techniques (PVS-VF, PVS-putty, and double-PVS group) and bracket geometry (with and without hook). Furthermore, the differences in maximum linear and angular deviations as well as excess adhesive were compared using the Tukey’s multiple comparisons post hoc tests using the statistical program Prism (version 6, GraphPad Software Inc., La Jolla, CA, USA). *P* ≤ 0.05 was considered statistically significant. All results are expressed as mean ± SD.

## Results

### Debonded brackets

Of 840 brackets, 11 remained in the trays after removal from the transfer model: 5 in the PVS-VF group [#11, #13, #19, #20, #29; 2 with hook (WH), 3 without hook (WOH)], 4 in the PVS-putty group (#3, #4, #27, #28; 2 WH, 2 WOH), and 2 in the double-PVS group (#3, #18, both WH).

### Measurements

**Fig. 10:**
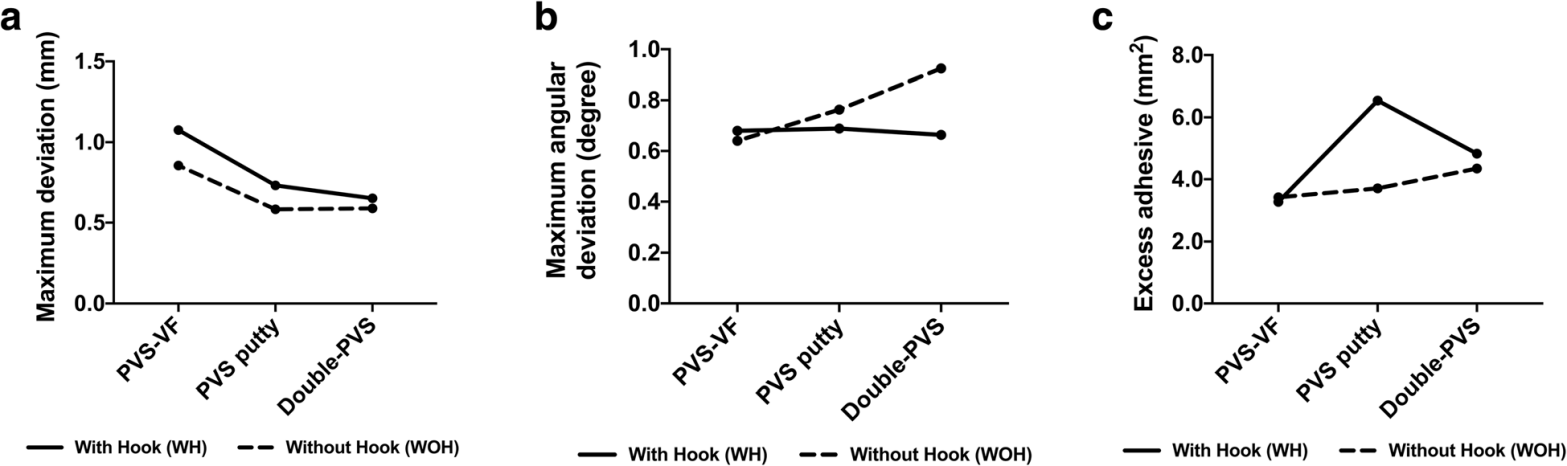
Interaction plots of possible interactions between bracket geometry (with hook, and without hook,) and the indirect bonding (IDB) technique (PVS-VF, PVS-putty, and double PVS) for **a** maximum bracket deviation, **b** maximum angular deviations, and **c** excess adhesive

**Fig. 11:**
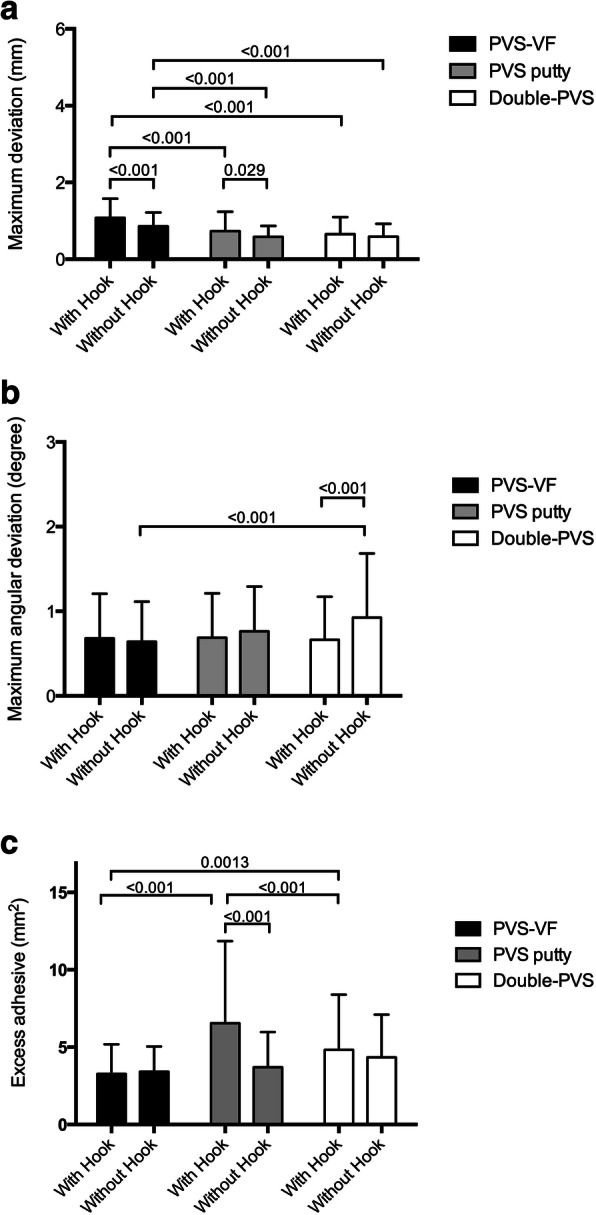
**a** Comparison of mean differences of maximum bracket deviation in bracket position among the three techniques depending on bracket geometry. *P* ≤ 0.05. **b** Comparison of mean differences of maximum angular deviation in bracket position among the three techniques depending on bracket geometry. *P* ≤ 0.05. **c** Comparison of mean differences of maximum excess adhesive after bracket position among the three techniques depending on bracket geometry. *P* ≤ 0.05

The maximum linear and angular deviations of the brackets as well as the excess adhesive depending on the IDB techniques (PVS-VF, PVS-putty, and double-PVS) and bracket geometry (WH and WOH) are shown in Table 2. Possible interactions between IDB technique and bracket geometry are shown in Fig. 10 and corresponding *P*-values are listed in Table 3. Figure 11 shows the comparisons of the mean values with corresponding *P*-values of bracket deviations and excess adhesive.

**Table 2:** Mean differences in bracket position between working and transfer models depending on bracket geometry

	Bracket	*N*	Maximum deviation (mm)				Maximum angular deviation (°)				Excess adhesive (mm^2^)			
			Mean	SD	Min	Max	Mean	SD	Min	Max	Mean	SD	Min	Max
PVS-VF	With Hook	118	1.08	0.50	0.31	2.38	0.68	0.53	0.01	2.07	3.27	1.91	0.00	7.67
	Without Hook	157	0.86	0.36	0.16	1.74	0.64	0.48	0.02	1.86	3.42	1.63	0.13	6.79
PVS putty	With Hook	118	0.73	0.51	0.21	2.24	0.69	0.52	0.02	2.31	6.54	5.31	0.24	22.55
	Without Hook	158	0.58	0.28	0.22	1.43	0.76	0.53	0.01	2.04	3.71	2.26	0.33	10.03
Double-PVS	With Hook	118	0.65	0.45	0.17	1.79	0.66	0.51	0.01	2.05	4.83	3.56	0.00	15.06
	Without Hook	160	0.59	0.33	0.12	1.58	0.92	0.76	0.01	3.05	4.35	2.76	0.00	11.13

**Table 3:** *p*-values of ANOVA about possible interaction between the indirect bonding technique and bracket geometry

	Maximum linear deviation	Maximum angular deviation	Excess adhesive
Interaction	0.074	0.008	0.001
Tray factor	< 0.001	0.014	< 0.001
Geometry factor	< 0.001	0.024	< 0.001

Statistically significant differences in maximum deviations were obtained between the PVS-VF (WH: 1.08, SD 0.50 mm; WOH: 0.86, SD 0.25 mm) and PVS-putty (WH: 0.73, SD 0.51 mm; WOH: 0.58, SD 0.28 mm) as well as the double-PVS group (WH: 0.65, SD 0.45 mm; WOH: 0.59, SD 0.33 mm) for comparisons WH and WOH (*P* < 0.001). Between the WH and WOH subgroups, significant differences were observed within the PVS-VF (*P* < 0.001) and PVS-putty group (*P* = 0.029).

Concerning the maximum angular deviation, significant differences between the groups were only observed between PVS-VF and double-PVS WOH (0.64, SD 0.48° vs. 0.92, SD 0.76°, *P* < 0.001) and for the comparison of brackets WH and WOH in the double-PVS group (WH: 0.66, SD 0.51° vs. WOH: 0.92, SD 0.76°, *P* < 0.001).

Highest excess adhesive values were determined after using the PVS-putty technique around brackets WH. Statistically significant differences were determined by comparing the excess adhesive between all IDB techniques using brackets WH: PVS-VF vs. PVS-putty (3.27 SD, 1.91 mm^2^ vs. 6.54 SD, 5.31 mm^2^, *P* < 0.001), PVS-VF vs. double-PVS (3.27 SD, 1.91 mm^2^ vs. 4.83 SD, 3.56 mm^2^, *P* = 0.0013), and PVS-putty vs. double-PVS (6.54 SD, 5.31 mm^2^ vs. 4.83 SD, 3.56 mm^2^, *P* < 0.001). No statistically significant differences were observed between the IDB techniques for brackets WOH. Within the subgroup, statistically significant differences were obtained in the PVS-putty group (WH: 6.54, SD 5.31 mm^2^ vs. WOH: 2.26, SD 0.33 mm^2^, *P* < 0.001). For all measurements, the mean variation was basically higher using brackets WH, except for the maximum angular deviation in the double-PVS group.

### Interactions between factors

Possible interactions between the type of IDB techniques and bracket geometry were observed for all collected parameters (Fig. 10), except between the type of technique and bracket geometry concerning the maximum deviation (Table 3).

With regard to the maximum deviations the mean values were similar of brackets WH and WOH for all types of IBD technique; however, with larger differences depending on IDB group. Less effect was caused by bracket geometry compared to IDB technique, especially using double-PVS (Fig. 10 A). Considering the maximum angular deviations, a significant interaction between bracket geometry and tray factor was found (*P* = 0.008). While the use of brackets WH and WOH combined with PVS-VF technique led to similar deviations in angulation, the combination with double-PVS technique conducted greater deviations for brackets WH. Ordinal effects for the bracket geometry existed between PVS putty and double-PVS, and a disordinal effect between PVS-VF and PVS putty. This means less deviation when using brackets WOH combined with PVS-VF technique (Fig. 10 B). Regarding the bonding adhesive most of excess occurs using brackets WH placed by PVS putty technique, and less interactions was observed using PVS-VF (Fig. 10 C).

## Discussion

Different techniques for the IDB of orthodontic brackets exist. These can differ in the material, which is used for the transfer tray or the type of bonding adhesive. Most of the studies deal with the transfer tray. However, next to the tray material the kind of bonding adhesive could affected in the results. Therefore, the present study investigates with the effects of three common IDB techniques based on three different siloxane trays and two different bonding adhesives for maximum linear and angular bracket deviation as well as compares and evaluates the excess bonding adhesive material around the bracket base; it also investigates if bracket hooks or tubes affect the mentioned parameters. To prevent tooth positioning effects, tooth shapes or crowding maxillary and mandibular typodont plaster models with ideally positioned teeth were used. Virtual working and transfer models were compared using automatic surface registration of the virtual models based on a completely automatic iterative closest point algorithm.

To avoid reflection from the brackets surface during the scanning process scan powder was used. But this powder can also lead to less accuracy during the scanning process due it has an average thickness of 28.6 μm and lead to a total increase error in the digitizing process about 48.8 μm [23]. Nevertheless, in a current investigation in prosthetic dentistry it was found that powder application before scanning improved the vertical fit of crowns and reduced the volumetric 3D internal fit [24]. Furthermore, the measured maximum deviations in the present study ranged between 0.58 SD 0.28 mm to 1.08 SD 0.50 mm. Thus, the powder can be neglected as an influencing factor on scanning accuracy and is without clinical importance regarding the necessary precision in bracket positioning.

Data on critical bracket placement discrepancies varies in currently available literature. Armstrong et al. assessed changes of ≥0.25 mm to upper central and lower incisor brackets and 0.5 mm to the remaining teeth as clinically significant [25], whereas the American Board of Orthodontics deducts points for alignment or marginal ridge discrepancies ≥0.5 mm and angular deviation ≥2° in their model grading system [26]. Castilla et al. also investigated bracket accuracy depending on different IDB trays. Because an additional misplacement on adjacent teeth of the same extent in an opposite direction would result in discrepancies ≥0.25 mm, a 0.13-mm discrepancy in any direction was defined to be clinically relevant [13].

Regarding maximum linear deviation, the highest discrepancy was observed in the PVS-VF tray followed by the PVS-putty, both combined with chemically cured adhesive and double-PVS trays combined with light cured adhesive; however, no differences were found between the PVS-putty and double-PVS group. These effects have been detected both for brackets WH and WOH. When brackets WH and WOH were compared, a larger deviation was obtained for brackets WH, except in the double-PVS group. Thus, the largest linear deviations can be expected using PVS-VF and brackets WH, while the use of PVS-putty and brackets WOH seem to allow ideal bracket placement; however, no statistical significance in the apparent interactions between IDB technique and bracket geometry was noted.

In contrast to the present study, Castilla et al. focused only on the influence of five different IDB trays measuring the mesiodistal, occlusogingival, and faciolingual bracket deviations and observed that bracket transfer accuracy was comparable for all silicone-based (double-PVS, PVS-VF, and PVS-putty) techniques [13]. In contrast, single- and double-vacuum-forms were significantly less accurate than silicone-based techniques in occlusogingival direction, but only for anterior teeth. Dorfer et al. reported similar results [14]. They observed the greatest inaccuracies of the vertical dimension. Comparing the values for maximum linear deviation of the present study with those already postulated in current literature as well as the permitted critical values, the values obtained in the present study are significantly higher for both cases. Possible reasons could be that bracket accuracy was measured as an overall deviation and not classified according to deviations in the three separate space planes; besides, the calculation was automatic, lessening the possibility of user errors.

Comparing the maximum angular deviations, significant differences were found only between the PVS-VF and double-PVS group. Likewise, differences in hook presence were observed only within the double-PVS group. Therefore, according to this parameter, the transfer material appears less important. Nevertheless, the interaction between the type of IDB technique and the bracket geometry seems to have an effect on the angular deviation.

Compareing the results of the present study to Shpack et al. less angular deviation but more linear deviation was found. Shpack et al. reported a torque error about 3.02° and rotation deviation about 0.75 mm [27]. The differences may be due in the different IDB techniques or the way of assesment. Shpack et al. used the preferable bracket placement technique by jigs and a manual assignment. In contrast, in this investigation the bracket transfer based on siloxane trays and the assignment was completely automated.

Grünheid et al. also investigated the transfer accuracy of vinyl polysiloxane trays for IDB [12]. Patients’ dental casts were scanned before and after bracket transfer using cone beam computed tomography to capture virtual positioning data and superimpose them digitally and determine the linear dimension, torque, and rotation. Clinically acceptable discrepancies were set according to the American Board of Orthodontics grading system (linear ≤0.5 mm, angular ≤2°) [26]. They reported no significant differences in individual bracket placement. The transfer accuracy was the lowest for torque (80.15%) and highest for mesiodistal and buccolingual bracket placement (both 98.53%). There was a modest directional bias toward the buccal and gingival surfaces. They finally concluded that IDB using VPS trays transfers the planned bracket position from the dental cast to the patient’s dentition with a generally high positional accuracy. In the present study, angular deviations were also included as critical values. Thus, linear deviation does not seem to significantly impact the bracket angle.

A common method for investigating excess adhesive around brackets is using a microscope combined with a corresponding computer measurement tool. For this purpose, the distance between the bracket edge and the most/least leaked adhesive margin was metrically registered. Multiple values for every bracket side must be measured for this technique; however, this only creates an estimate and does not perform an exact measurement. In the present study, for the first time, excess bonding adhesive was measured in three-dimensions depending on the IDB technique and bracket geometry. With regard to the excess adhesive, there were particular differences in the extent of the brackets WH depending on the IDB technique. The largest excess bonding material area was observed in brackets WH in the PVS-putty group followed by the double-PVS and PVF techniques. Between brackets WH or WOH, significant differences were observed only in the PVS-putty group. This is probably due to the fact that the hooks are not completely enclosed by the putty material and subsequently space for the adhesive resulted. Furthermore, it has to be taken into account that differences in the adhesive systems between PVS-VF or PVF putty and double-PVS existed and in the double-PVS group a low viscosity composite was slightly used. This could have had an impact on the flow properties in context of the entire bonding adhesive. However, the present results suggest no influence due the excess adhesive was the largest in the PVS group even no low viscosity composite was used here. Therefore, it can be assumed that both IDB technique and bracket geometry influence the placement accuracy; however, the combination of brackets WH and PVS-putty as the tray material leads to particularly large excess bonding adhesive area, which probably increases the risk of caries or white-spot lesions.

## Conclusions

Although the precision of the indirect bonding techniques of vinyl polysiloxane trays is already very high, there are significant differences between various materials, which can be additionally enhanced by the presence of hooks on the brackets. In this in-vitro study, the double-PVS group exposed promising results with respect to transfer accuracy, whereas the trays in the PVS-VF group offered least excess bonding adhesive and the highest bracket placement accuracy was generated in the PVS group. In this context it should be noted that PVS-putty is the easiest to handle with and also the cheapest, but leads to large excess bonding adhesive, especially in combination with hooked brackets or tubes. Regarding the present of hooks, those braces seem to be disadvantageous for IDB as they lead to lower precision and higher excess bonding adhesive. Further studies must show whether tooth positioning effects, tooth shapes or crowding, which must be expected in clinical practice will increase the measured transfer inaccuracies or might level off.

## Data Availability

All data generated or analysed during this study are included in this published article.

## Abbreviations

IDB: Indirect bonding
PVS: Polyvinyl siloxane
VF: Vacuum-formed
PETG: Polyethylene terephthalate glycol
WH: With hook
WOH: Without hook
